# Native Multidimensional Protein Complex Identification and Topology Characterization (Native MudPIT)

**DOI:** 10.1021/acs.analchem.6c01693

**Published:** 2026-07-20

**Authors:** Zihao Qi, Fei Fang, William J. Moeller, Guijie Zhu, Qianjie Wang, Qianyi Wang, Guangyao Gao, Liangliang Sun, Vicki H. Wysocki

**Affiliations:** † School of Chemistry & Biochemistry, 1372 Georgia Institute of Technology, Atlanta, Georgia 30332, United States; ‡ Native MS Guided Structural Biology Center, Georgia Institute of Technology, Atlanta, Georgia 30332, United States; § Department of Chemistry, 3078 Michigan State University, East Lansing, Michigan 48824, United States

## Abstract

The initiation and progression of diseases are usually due to biological processes going awry, driven by proteins in complexes that often assemble via noncovalent interactions of monomeric or multimeric subunits. A broader view of protein complex dynamics in cells is vital for advancing our understanding of the molecular mechanisms of diseases. Here, we present a novel mass spectrometry (MS)-based native proteomics technology, native **mu**lti-**d**imensional **p**rotein complex **i**dentification and **t**opology characterization **(Native MudPIT)**, to achieve proteome-scale protein complex characterization. Native MudPIT integrates native size exclusion chromatography (SEC), capillary zone electrophoresis-MS (nCZE-MS), surface-induced dissociation (SID), and multilevel proteomics. The technique enabled the detection of 124 endogenous proteins/protein complexes with masses up to 1.5 MDa from an *Escherichia coli* whole cell lysate, identified 46 protein complexes in a discovery mode, and provided topological information on 39 protein complexes. This work represents a pivotal advance in native proteomics for global and comprehensive characterization of protein complexes in complex biological systems (i.e., cells, tissues, and biological fluids).

## Graphical abstract

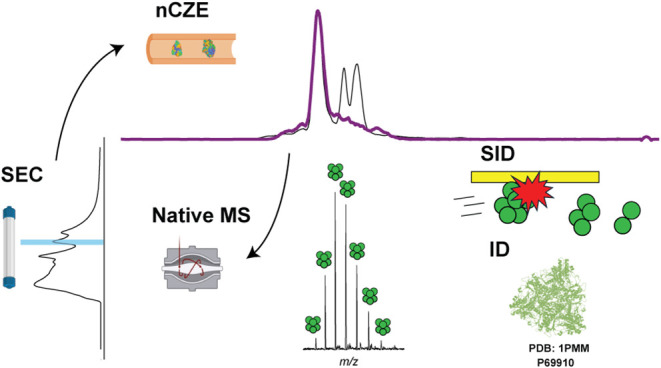

## Introduction

Diseases are usually caused by biological processes going awry, which is driven by proteins in complexes that often assemble via noncovalent interactions of monomeric or multimeric subunits.^1,2^ A deep view of protein complex dynamics in cells is vital for advancing our understanding of the molecular mechanisms of diseases. Mass spectrometry (MS)-based proteomics has been widely used to profile protein–protein noncovalent interactions in discovery mode and on a proteome scale using different techniques, including affinity purification-MS (AP-MS), proximity labeling-MS (PL-MS), cross-linking-MS (XL-MS), cofractionation-MS (CF-MS), thermal protein profiling (TPP)-MS, and limited proteolysis (LiP)-MS.^3^ These MS-based proteomics strategies have substantially advanced our understanding of protein–protein interaction dynamics in cellular processes. However, these approaches rely on bottom-up proteomics, which measures proteolytic peptides and builds protein-level information from the identified peptides, failing to offer details of specific proteoforms that form protein complexes and only providing indirect measurements of protein complexes, losing a comprehensive view of protein complexes.^4^

Native mass spectrometry (nMS) has been widely used for the direct measurement of purified protein complexes up to megadaltons (MDa) in size.^5–7^ State of the art nMS technology can transfer various macromolecules into the gas phase gently enough that information at the protein complex level can be maintained, such as the subunit stoichiometry and topology. Native MS-based native structural proteomics aims to characterize the features of endogenous proteins and protein complexes, including their solution equilibrium, oligomeric assemblies, and ligand-binding properties, in cells, tissues, and biological fluids, effectively and directly on a proteome scale under near physiological conditions.^2,8–10^

Native structural proteomics can be operated under the typical direct infusion mode.^11–17^ However, pre-MS separations or fractionations are essential to simplify the complexity from proteome-scale mixtures and for the measurement of relatively low-abundance protein complexes in complex samples.^18^ Native capillary zone electrophoresis (nCZE),^8,9,19–23^ native capillary isoelectric focusing,^24^ size exclusion chromatography (SEC),^15^ and other separation techniques,^10,18,25,26^ followed by multistage MS/MS, were recently developed to define the stoichiometry of oligomeric assemblies and identify protein complexes and post translational modifications (PTMs) from complex mixtures. The size of protein complexes that can be measured from complex samples is, however, still limited, and more importantly, the current native proteomics techniques do not provide structural information (i.e., topology) of protein complexes, which could more directly link to their functions.

In this work, we introduce a novel method, native **mu**lti-**d**imensional **p**rotein complex **i**dentification and **t**opology characterization **(Native MudPIT)**,^27,28^ that integrates multidimensional separation with surface-induced dissociation (SID) as an effective MS/MS tool for protein complex identification and topology characterization (SEC-nCZE-SID-MS). Multilevel proteomics (bottom-up, top-down, and native proteomics) was employed to facilitate the identification and characterization of protein complexes. SID involves a rapid, high-energy deposition, cleaving the noncovalent interfaces of protein complexes. By keeping each subcomplex intact, SID generates structurally informative fragments.^29^ When coupled with multidimensional separation, SID enables the interrogation of protein structures from complex mixtures, as demonstrated by an *Escherichia coli* cell lysate model system in this work.

## Materials and Methods

### Materials and Chemicals

All reagents, such as Dulbecco’s phosphate-buffered saline (DPBS) and Amicon Ultra centrifugal filter units (0.5 mL, 30 kDa cutoff), were obtained from Sigma-Aldrich (St. Louis, MO) unless stated otherwise. Protease inhibitors (complete ULTRA Tablets) and phosphatase inhibitors (PhosSTOP) were from Roche (Indianapolis, IN). LC/MS-grade water, acetic acid, formic acid, methanol, and ammonium acetate, as well as hydrofluoric acid (HF), were purchased from Fisher Scientific (Pittsburgh, PA). Bare fused silica capillaries (50 μm i.d., 360 μm o.d.) were purchased from Polymicro Technologies (Phoenix, AZ), and acrylamide was purchased from Acros Organics (Fair Lawn, NJ).

### Sample Preparation

*E. coli* (strain Top10) was cultured in Terrific Broth (TB) medium at 37 °C with shaking until the optical density at 600 nm (OD_600_) reached 0.77. The bacterial cells were then washed three times with DPBS and pelleted by centrifugation. A 0.45 g pellet was resuspended in 1.6 mL DPBS supplemented with complete protease inhibitors and phosphatase inhibitors (1%, w/v) and homogenized for 30 s three times using homogenizer 150 (Fisher Scientific) and subsequently sonicated on ice using a Branson Sonifier 250 (VWR Scientific, Batavia, IL) for 2 min, repeated 6 times. The lysate was centrifuged at 16,000*g* for 20 min at 4 °C, and the resulting supernatant was collected. Protein concentration (∼6 mg/mL) was determined using the bicinchoninic acid (BCA) assay (Fisher Scientific).

### Size-Exclusion Chromatography (SEC) Fractionation

Lysates were subsequently injected (20 μL per injection) into an Agilent Infinity II HPLC system equipped with an Agilent Bio SEC-5 column (4.6 mm × 300 mm, 5 μm particle size, 500 Å pore size) from Agilent Technologies (Santa Clara, CA). The mobile phase was 100 mM ammonium acetate, pH 7 at a flow rate of 0.2 mL/min. Fractions were collected every minute from 8.5 to 23.5 min, while the sample was collected from 23.5 to 27 min as one fraction, yielding a total of 16 fractions. Corresponding fractions from 16 replicates were combined and concentrated using Amicon-30 kDa centrifugal filter units. The buffer was exchanged by washing twice with 20 mM ammonium acetate (pH 6.9). The proteins were collected, and the concentration was measured using the BCA assay. The recovered protein samples were frozen in −80 °C until analysis.

### Native Capillary Zone Electrophoresis–Mass Spectrometry (nCZE-MS)

Capillary zone electrophoresis (CZE) experiments were carried out using a Beckman CESI8000 Plus capillary electrophoresis autosampler (SCIEX, Framingham, MA). A commercial electrokinetically pumped sheath flow nanospray source (EMASS-II, CMP Scientific, Brooklyn, NY) was used to interface the CZE platform with a Q-Exactive Ultra-High Mass Range (UHMR) Orbitrap mass spectrometer (Thermo Fisher Scientific, Waltham, MA), which was modified with a 3 cm, 12-lens surface-induced dissociation (SID) device. The nanoelectrospray ionization (nanoESI) emitters used in the EMASS-II interface were pulled from borosilicate glass capillaries (1.0 mm o.d., 0.75 mm i.d.) using a Sutter P-1000 Flaming/Brown pipet puller (Sutter Instrument, Novato, CA), resulting in an orifice size of 25–35 μm. The voltage of nanoESI was set to 2 kV and kept consistent across all experiments. To avoid electroosmotic flow, the capillaries used in all experiments (50 μm i.d., 360 μm o.d.) were coated with neutral linear polyacrylamide (LPA). One end of the coated capillary was etched with HF at the end near the CE-MS interface to reduce the outer diameter of the capillary. The detailed preparation procedure was based on our previous protocol.^30–32^ For nCZE-MS and nCZE-MS/MS experiments, a ∼85 cm LPA-coated capillary was used for separation. A high voltage of +20 kV and an assisting pressure of 1 psi were applied for a 50 min separation, followed by 20 psi pressure applied for 15 min to flush the capillary. Sample injection into the capillary was performed using 5 psi air pressure, with 50 nL of ∼1 mg/mL sample injected, calculated based on Poiseuille’s law. The background electrolyte (BGE) consisted of 20 mM ammonium acetate, while the sheath liquid contained 20 mM ammonium acetate.

All MS settings were kept the same unless specified: Source temperature: 250 °C; Source DC offset: 21 V; S-lens RF level: 200; in-source trapping (IST) desolvation voltage: −60 V for MS1 collection while −30 V for others; Injection Flatapole, Inter Flatapole, and Bent Flatapole were set to 5, 4, and 2 V, respectively; resolution: 6250 defined at 400 *m*/*z*; AGC target: 1 × 10^6^; maximum injection time: 200 ms; mass range: 1000–23,000 *m*/*z*; C-trap Entrance: 1.8 V and C-trap Exit: 16 V, trapping gas setting: 5 with a HV sensor read back roughly 10^–9^ mbar. Trapping gas settings for HCD experiments were set to 3 for better detection of unfolded monomers.

### Native CZE-MS/MS and All Ion Fragments

SID MS/MS was performed using a custom SID device described previously.^33^ Ion activation was performed by keeping the SID voltages constant for the entire fraction. For each fraction, two SID voltages, 65 and 125 V, were used to provide complementary structural information. Voltages applied to all 12 lenses were controlled using an external power supply (Ardara Technologies, Ardara, PA) at values listed in Table S1. HCD MS/MS was performed in a similar manner, with HCD at 200 V kept constant throughout the run.

### CZE-Based Top-Down Proteomics

Top-down proteomics experiments on denatured protein were performed using a Beckman CESI8000 Plus capillary electrophoresis system coupled to a Thermo Exploris 480 Orbitrap mass spectrometer via the EMASS-II interface. A 90 cm LPA-coated capillary (50 μm i.d., 360 μm o.d.) was used for separation. The BGE consisted of 5% acetic acid (v/v), while the sheath liquid contained 0.2% formic acid and 10% methanol (v/v).

For CZE separation in top-down proteomics, a high voltage of +30 kV was applied for 50 min, followed by 10 min of flushing method with +30 kV and 20 psi forward. Sample injection was carried out using 5 psi air pressure, with an injection volume of 50 nL, calculated based on Poiseuille’s law. Two mass spectrometry acquisition modes were employed: low-high and high–high.

In low-high mode, MS1 acquisition was performed at a resolution of 7500 with a 300% normalized AGC (automatic gain control) target and five microscans. The *m*/*z* range was 600–3000. The maximum injection time was set to auto, with a minimum intensity threshold of 1 × 10^4^. Charge states ranging from +5 to +60, along with undetermined charge states, were included. Dynamic exclusion was enabled after three occurrences, with an exclusion duration of 30 s. The six most intense ions were selected for MS/MS, using an isolation window of 4 *m*/*z*. Fragmentation was performed using stepped higher-energy collisional dissociation (HCD) at 25%, 35%, and 45%. MS2 acquisition was conducted at a resolution of 240,000, and the *m*/*z* range was 200–2000. The normalized AGC target was set as 100% with a single microscan. The maximum injection time remained in auto mode.

In high–high mode, a 15 V source fragmentation was applied. MS1 acquisition was performed at a resolution of 480,000, with a 300% normalized AGC target and a single microscan. The isolation window was set as 2 *m*/*z*, and MS2 acquisition was conducted at a resolution of 120,000. Other acquisition parameters were used as in the low-high mode, except that the undetermined charge was not included in the top 6 selection for MS/MS.

### CZE-Based Bottom-Up Proteomics

For each SEC fraction, 1 μL of 100 mM Dithiothreitol (DTT) was added to 5 μL of the sample and incubated at 70 °C for 10 min. Then the proteins were alkylated by adding 1 μL of 250 mM iodoacetamide (IAA) at room temperature for 30 min in the dark. Finally, the proteins were digested by trypsin at a trypsin/protein ratio of 1/30 (w/w) overnight at 37 °C. The digests were stored in −80 °C for further analysis.

For bottom-up proteomics, the CZE-MS instrument setup was the same as that for top-down proteomics. A 90 cm LPA-coated capillary (50 μm i.d., 360 μm o.d.) was used for separation, and 100 nL of each sample was loaded for analysis. For CZE separation, a high voltage of +30 kV was applied for 60 min, followed by 10 min of flushing method with +30 kV and 20 psi forward.

For the mass spectrometer, experiments were conducted in Data-dependent acquisition (DDA) mode. Full MS scans were acquired in the Orbitrap mass analyzer over the *m*/*z* 300–1500 range with a resolution of 60,000 (at 400 *m*/*z*). The 15 most intense peaks with a charge state of 2–6 were fragmented in the higher-energy collisional dissociation (HCD) cell with collision energy of 28% and analyzed by the Orbitrap mass analyzer with a resolution of 30,000 (at 200 *m*/*z*). The intensity threshold was 5.0 × 10^4^, the isolation window was 2 *m*/*z*, and the dynamic exclusion duration was 15 s. Normalized AGC target on MS1 was 300%, normalized AGC target on MS2 was 100% and a single microscan for both MS1 and MS2.

### Data Analysis

For the nCZE-MS and nCZE-MS/MS data, manual integration on the electropherograms was performed for each fraction to generate its corresponding mass spectra. Mass spectra were either deconvolved manually or using Unidec.^34^ The processed masses were compiled into Supporting Information II. Target masses were generated by inputting the IDs from the bottom-up experiments into the ID mapping function from UniProt, and the output was input into ProSight Native as the target database. HCD/SID-generated monomer masses were batch added to the complex-down function in ProSight Native for database searching.^35^ Higher-order oligomers were assigned based on the integer multiple of the monomer mass, based on the observed oligomeric state from the Protein Data Bank.

For all the TDP mass spectra obtained with high–high mode (high resolution MS1, high resolution MS/MS), proteoform identification from *E. coli* lysate was performed using TopPIC (Top-down mass spectrometry-based Proteoform Identification and Characterization) pipelines.^36^ Briefly, the raw file was converted to mzML files by MSConvert^37^ using the peak-picking algorithm. Then, the spectral deconvolution was performed using Top-down mass spectrometry Feature Detection (TopFD, version 1.6.3) with default parameters.^38^ The database search was performed using TopPIC (1.6.3) against the UniProt proteome database of *E. coli* (UP000002311, 4602 entries, accessed on 04/11/2025). The parameters were set as follows: mass error tolerance of matching masses of 10 ppm, maximum number of variable modifications (including acetylation, phosphorylation, oxidation, and methylation) of 3, maximum number of mass shifts of unknown modification of 2, ranging from −500 to 500 Da. The false discovery rates (FDRs) were estimated using the target-decoy approach. The spectrum level FDR cutoff was 1%, and the proteoform level FDR cutoff was 1%.

For complex samples analyzed by low-high mode, the raw files were first converted to mzML files by MSConvert using a peak-picking algorithm. Then, the spectral deconvolution was performed using TopFD, version 1.6.3, with the default parameters except that the maximum charge setting was 100, the maximum mass was 100,000 Da and enable Missing MS1 spectra. Then, the obtained MSAlign files were processed using ProSight Lite software.^39^

For the bottom-up experiment, the raw files were processed using SEQUEST integrated in Proteome Discoverer 2.2. The MS/MS spectra were searched against the UniProt *E. coli* proteome database (UniProt-proteome_UP00000625, 4588 entries, accessed on 04/17/2025). The search parameters were specified as precursor mass tolerance was 20 ppm; fragment mass tolerance was 0.02 Da. The maximum missed cleavages per peptide was 2. Carbamidomethylation of cysteine residues (+57.0215 Da) was set as a fixed modification. Acetyl (+42.011 Da­(N-Terminus)) was set as a dynamic modification on the N-terminal of the protein. Oxidation (+15.995 Da­(M)) and deamidation (+0.984 Da (N, Q)) were set as dynamic modifications. The target FDR of the decoy database search was set as 0.01.

## Results and Discussion

Figure 1 illustrates the workflow of this method. The *E. coli* cells were lysed in Dulbecco’s Phosphate-Buffered Saline (DPBS) and fractionated by offline SEC. Each fraction was buffer-exchanged into 20 mM ammonium acetate (pH 6.9) and sequentially subjected to online nCZE-MS separations followed by two types of gas-phase fragmentation techniques, SID and higher-energy CID (HCD). SID is highlighted here to probe the topology of the intact, multisubunit protein complexes by fragmenting them into subcomplexes. In contrast, HCD generated the unfolded monomers and (n-1)-mer. Based on the mass relationship between precursors and products, HCD and SID both offer information, including oligomeric states and monomeric masses, for identification. In our study, SID was used for protein complex identification and substructure/topology characterization, while the HCD-generated monomer masses were used to assist SID-based identification and, occasionally, were used directly to identify protein complexes. The detailed voltage settings for SID are shown in Table S1 in Supporting Information I.

**Figure 1:**
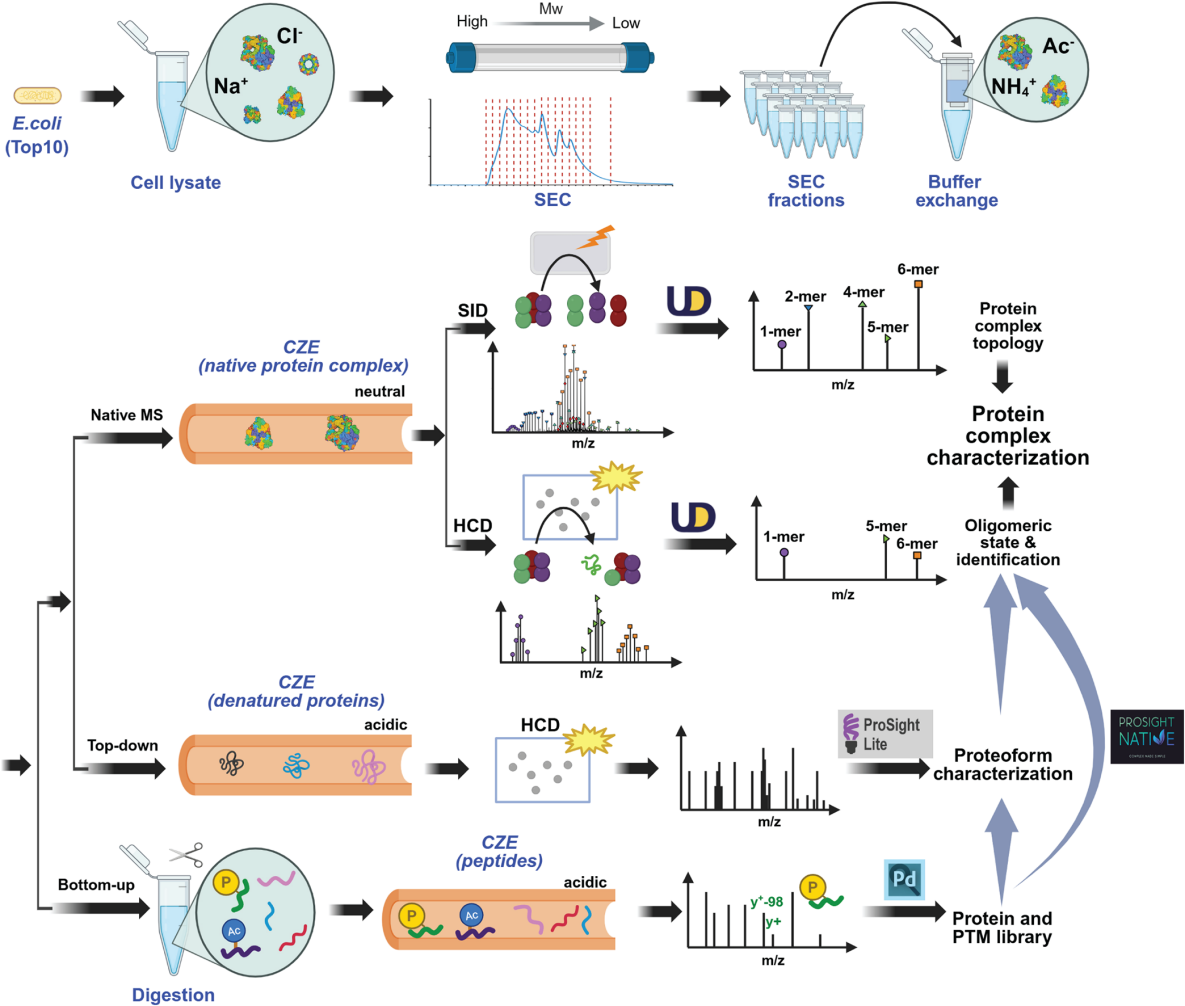
Schematic workflow of Native MudPIT. The *E. coli* cell lysate was fractionated with SEC, buffer-exchanged to 20 mM ammonium acetate, and partitioned for parallel CZE-MS-based analysis. Each SEC fraction was analyzed by nMS with MS1, SID, and HCD. Additional denatured top-down proteomics and bottom-up proteomic analyses were performed.

Because CZE only requires a small amount of sample, additional parallel experiments, including CZE-based denatured top-down and bottom-up proteomics, were also performed to both shrink the database to search against and characterize the protein at both the intact monomer (proteoform) and peptide levels. These additional experiments enhance identification of protein complexes, not only showing potential for information on PTMs and their locations but also simplifying the annotation process in native experiments. Specifically, monomer masses generated by either HCD or SID were imported into the ProSight Native^35^ complex-down algorithm and were searched against the database generated by the non-native experiments. Using the protein group generated by non-native experiments substantially reduced the mismatch compared with using the entire *E. coli* proteome database. As shown in Supporting Information II, the SID- and HCD-generated monomer masses used for identification have a median error of 1 Da from the expected values. Mass errors can vary at the complex level due to adducts, ligands, potential covalent modifications, or sequence variations (with the final two still distinguishable by monomer masses). However, accurate mass measurement at the monomer level still provides a strong mass constraint to support complex assignments. Additionally, oligomeric states and topologies were also used to evaluate the confidence of identification.

The multidimensional separation platform (SEC-nCZE-MS) produced efficient separations of large proteins/protein complexes. SEC reduces sample complexity through size-based fractionation, while CZE provides additional separation capacity within each fraction, Figure 2. Each 1 min SEC fraction was further separated by nCZE into a 10 min separation window (5–16 min shown in Figure 2) with about 4–6 broad electrophoretic peaks clearly visible on average. In the averaged mass spectrum from one electrophoretic peak, clear signals of multiple large proteins/protein complexes (i.e., 478 kDa, 669 kDa, and 861 kDa) were observed. Although the absolute peak capacity is modest compared to peptide-scale separations,^40^ it represents a meaningful multidimensional separation for native protein complexes, where resolution is inherently constrained by broad separation peaks. We note here that SEC prefractionation successfully reduces sample complexity; however, adjacent fractions contain substantial overlap (perhaps partially the result of overloading the column). Thus, we primarily analyzed nonadjacent fractions for CZE-MS/MS. Nevertheless, overlapping species were still detected across different fractions (Figure S1). The *m*/*z* dimension is treated separately as an additional source of orthogonal analytical discrimination. Our workflow highlights a substantial analytical space for detecting native protein complexes. The heatmap of the detected large proteins/protein complexes suggests the efficient separation of proteins by SEC in a mass range of 50–1450 kDa by their size under the native conditions, with the larger protein complexes eluting from the SEC column earlier, as expected.

**Figure 2:**
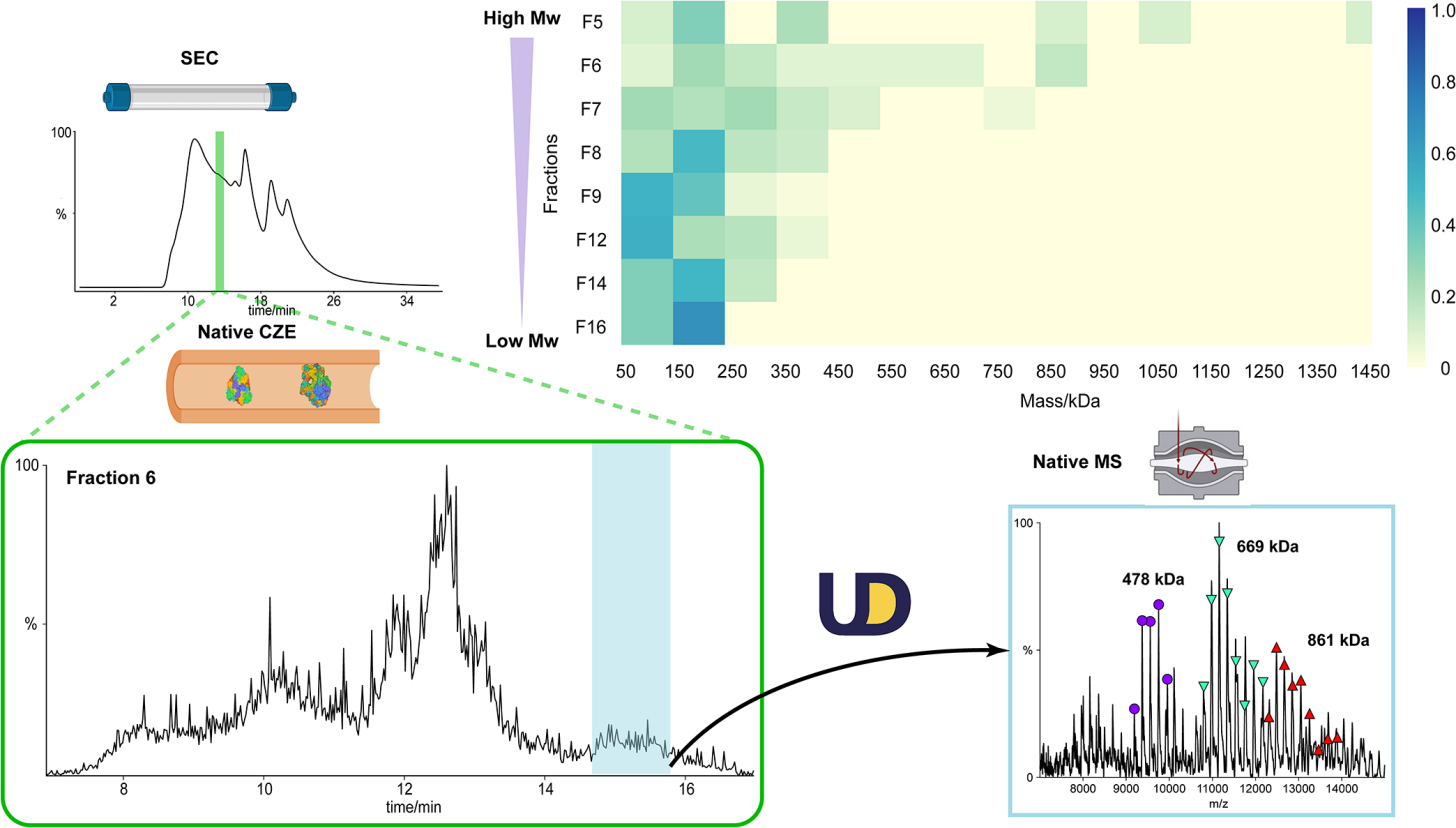
SEC chromatogram of the *E. coli* cell lysate, an electropherogram of one SEC fraction (Fraction 6) after nCZE-MS analysis with a highlighted mass spectrum of some large proteins/protein complexes (478 kDa, 669 kDa, and 861 kDa) after UniDec deconvolution,^34^ and a heat map showing the distributions of observed masses of protein/protein complexes as a function of SEC fraction number. Measured masses were binned every 50 kDa. The intensities of each fraction (row) in the heatmap were normalized such that the total number of protein complexes in each fraction (row) was set to 1.0. Each fraction (row) was normalized independently. Note that there are unresolved species spanning high *m*/*z* regions, and only well-resolved charge state envelopes were used to generate this heatmap.

In total, 124 proteins/protein complexes with masses over a range of less than 10 kDa to approximately 1.5 MDa were detected. The measured 1.4 MDa species from fraction 5 is shown in Figure S2. Compared with our previous native CZE-MS data of an *E. coli* cell lysate,^8^ Native MudPIT expands the accessible mass range and increases the number of detectable protein complex species relative to prior reports in the context of LC/CE measurements.^41,42^ The detected protein complexes’ masses from each SEC fraction are listed in Supporting Information II. The high separation capacity of our native SEC-CZE-MS platform not only boosted the detectable proteome scale but also reduced the sample complexity for MS measurement, facilitating the interpretation of the following MS/MS measurements for protein complex identifications and topology characterization.

With our Native MudPIT technique, we identified 46 protein complexes, with 39 identified by SID together with their topology and oligomeric state, 36 identified by HCD, and 28 identified by both fragmentation methods at the intact mass level. In most cases, the SID-generated monomer and HCD-generated monomer both match the sequence mass, with one example shown in Figure S3. In addition to producing structurally informative fragments, SID offers an advantage due to its more symmetric charge partitioning, which keeps product ions within a relatively narrow *m*/*z* range. By contrast, HCD products often fall into widely separated *m*/*z* regions, leading to uneven detection or loss of the complementary ion. Using mass balance, the relationship between the ejected unfolded monomer and the intact precursor can be sufficient for assignment in simple homo-oligomers. However, HCD may limit direct product-pair confirmation, especially for heteromeric complexes, where direct observation of complementary products improves confidence in the composition annotation.

The identification of proteoforms forming the protein complexes is at the fifth level^43^ by nMS with HCD or SID, where only the accurate monomeric mass of the proteoform is known. However, given that those proteins making up the complexes were also identified from the parallel top-down and bottom-up experiments of the same SEC fractions, this served as a validation and improved the level of proteoform characterization to levels 1 to 3, where the PTMs and their locations can also be determined. We note that only a fraction of the detected protein complexes were identified, most likely due to the relatively low MS signal of many detected protein complexes, leading to difficulties in producing sufficient HCD/SID fragment ions for identification. The lists of identified protein complexes are shown in Supporting Information II. The lists of identified proteoforms and protein groups from denaturing top-down proteomics and bottom-up proteomics of each SEC fraction are also shown in Supporting Information II. Compared to previous work showing a group of highly expressed protein complexes exhibiting dynamic oligomeric distributions,^44^ we detected all by bottom-up proteomics, whereas approximately half were identified by top-down proteomics and one-fourth by native MudPIT. This trend aligns with the overall number of protein groups identified by each method. As an additional proteomics-style annotation overview, identified proteins were classified using PANTHER (Figure S4).^45^ The resulting charts summarize the broad protein classes represented by each workflow.

The identification of several native complexes from the SEC-CZE-SID/HCD-MS data are presented here as representative of what is possible in this experiment. The first case shows a mass of approximately 203 kDa, centered around a 31+ charge state in MS1 (Figure 3a). With low and high energy SID, half of and one-fourth of the mass and charge were observed, respectively, suggesting the oligomeric complex is a tetramer with a dimer-of-dimers topology. By comparing the monomer mass with the high-abundance protein list from the bottom-up proteomics data of the same SEC fraction, either manually or via ProSight Native, the protein complex was identified as pyruvate kinase I tetramer, which agrees with the oligomeric assembly and topology of this protein complex in the literature.^46^ Pyruvate kinase plays vital roles in modulating the metabolic flux and ATP production,^47^ and has been connected to many diseases, including cancer,^48^ immune,^49^ and cardiovascular diseases.^50^ The capability of our technique to identify and characterize the endogenous pyruvate kinase complex within a complex biological system opens the door to a wide range of functional studies of this and other critical protein complexes in regulating fundamental cellular processes and disease progression.

**Figure 3:**
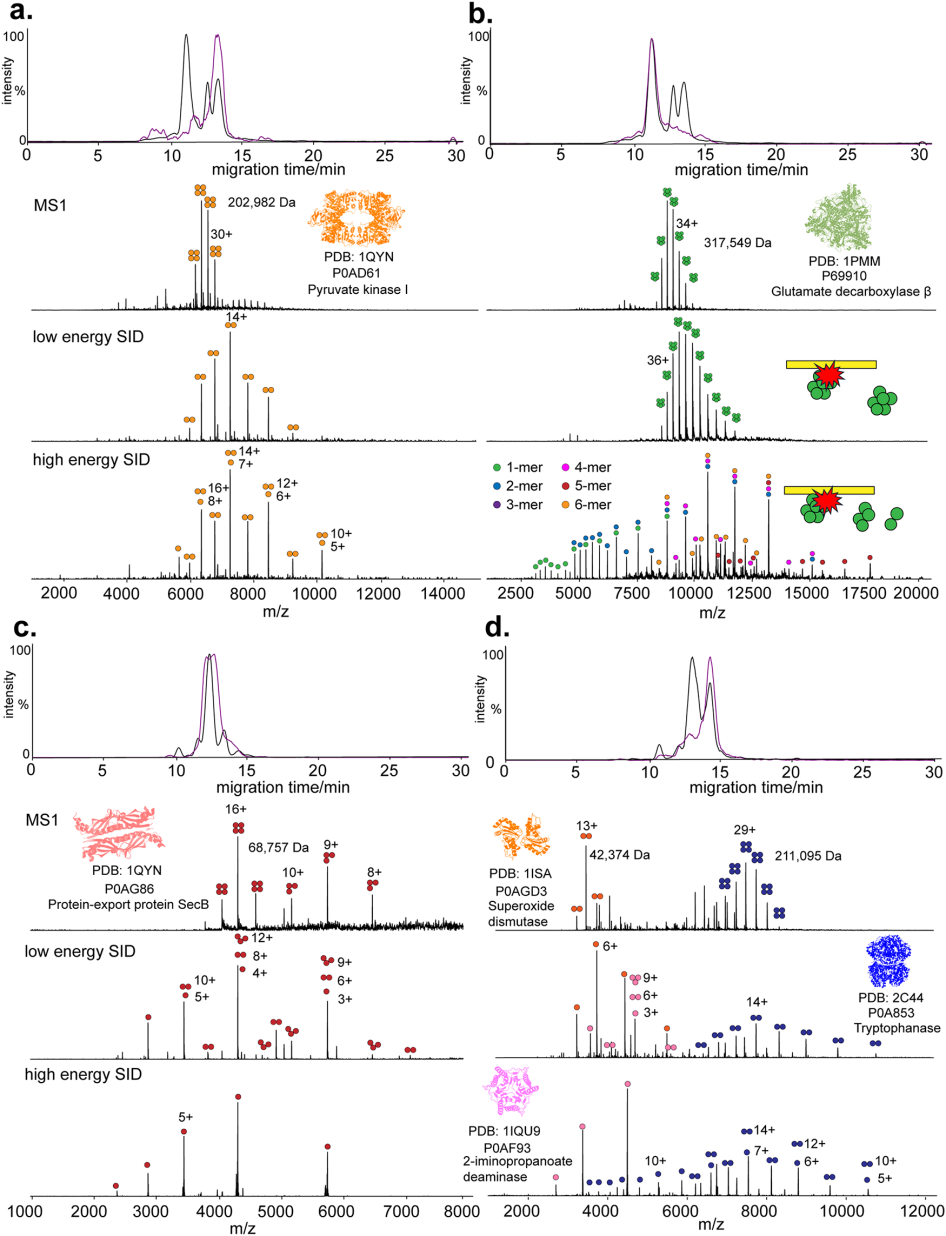
Highlighted examples identified by the SEC-nCZE-SID-MS workflow. For each subfigure (a-d), the top panel represents the overlay of the extracted ion electropherogram (purple) for a specific protein complex and its total ion electropherogram (black). MS1, SID 65 V, and SID 125 V are shown from the top to the bottom panel below the electropherogram, as labeled in each row.

The complex in Figure 3b shows an observed mass around 317 kDa, centered around 35+ in MS1. With low SID energy, the signal-to-noise ratio improved, but no significant fragmentation was observed. At high SID energy, this protein complex displays a characteristic fragmentation pattern, with fragments indicating a hexameric precursor because mostly dimer and tetramer fragments, and relatively low abundance of monomer and pentamer were produced; interestingly, almost no trimer fragments were observed. This fragmentation pattern suggests this hexameric protein to be a trimer of dimers topology, where the interfaces mediating three dimers are equally weak. Although the measured HCD/SID-generated monomer mass (Figure S5) does not match any sequence masses in the database close enough for direct identification, using the oligomeric state and topological information, this protein complex was identified as glutamate decarboxylase hexamer. There are two highly similar glutamate decarboxylase genes in *E. coli*, with a high degree of sequence homology (99%). Due to the high level of similarity between forms, we believe the detected species is a mixture of the two. Glutamate decarboxylase converts glutamate to γ-aminobutyrate (GABA) and plays critical roles in bacterial acid resistance and diabetes.^51–54^ At the monomer level, it binds pyridoxal phosphate (PLP) covalently (+229 Da) to give an overall expected monomer mass of 52,914 Da and 52,897 for α and β respectively, compared with our experimental mass of 52,902 Da. The widths of the peaks in the *m*/*z* space (∼1.5 *m*/*z*) are sufficiently broad to encompass both species. The experimental monomer masses generated by HCD and SID are the same, suggesting the endogenous ligand is covalently attached or very tightly bound.

In the third “unknown” case, a mass of approximately 68 kDa was observed, centered around a 16+ charge state in MS1 (Figure 3c). With low SID energy, multiple subcomplexes are generated, while with high SID energy, only a compact monomer is generated. The observed monomer mass was one-fourth the mass of a species observed in MS1, strongly indicating that the observed species is a tetramer. The observed fragmentation pattern suggests either a ring-shaped protein complex or a dimer-of-dimers protein complex, whose interface between two subunits, localized diagonally, is extremely strong. Based on the monomer mass and oligomeric state, this protein complex has been identified as the Protein-exported protein SecB, a chaperone dedicated to protein translocation. To further confirm our evaluation, protein interface analysis by PISA (Protein Interfaces, Surfaces and Assemblies)^55^ was also performed, as we have previously described,^29^ and the fragmentation patterns match remarkably with the calculated interfaces. The smallest total interface area needed to generate dimer fragments is 1820 Å^2^ (550 Å^2^ × 2 + 360 Å^2^ × 2 = 1820 Å^2^), and the total interface area needed to generate a monomer with its complementary trimer is 1900 Å^2^ (550 Å^2^ + 990 Å^2^ + 360 Å^2^ = 1900 Å^2^). The amount of interfacial area needed to generate monomer plus trimer versus dimer plus dimer is almost the same, matching our experimental results.

All three examples listed above are well-separated by nCZE, as is evidenced by the extracted ion electropherograms shown in Figure 3a–c. There are also instances where multiple protein complexes comigrate, even averaging a small slice of the window in the electropherogram, which is shown in Figure 3d. This case highlights three protein complexes that comigrate with each other but are identified. The dominant observed species has a mass of ∼211 kDa centered around the 29+ charge state in MS1, with low energy SID generating species with half the mass and high SID energies generating species with one-fourth of the mass, suggesting a tetramer with dimer-of-dimers topology. With the monomer mass, oligomeric state, and structural configuration, this protein complex is identified as tryptophanase. Another high-abundance mass species has a mass of around 42 kDa centered around a 13+ charge state. With low SID energy, a 21 kDa fragment with approximately half the charge was observed, and no further fragmentation was observed at high energy, implying that this protein complex is a dimer and is identified as superoxide dismutase. In the same window, another trimeric protein complex was identified by using only low and high SID energy, where low SID energy displays subcomplexes with 2/3 and 1/3 of the precursor mass, but high SID energy generates only monomers. With the information obtained from SID, assisted by bottom-up proteomics data, this protein complex was identified as the 2-iminopropanoate deaminase trimer. The results shown here further highlight the ability of our native MudPIT approach to characterize protein complexes in composite samples, enabling high-throughput functional studies of many complexes.

Multiple works have demonstrated the technical reproducibility of the CZE-based proteomics workflow, either native or non-native.^8,56,57^ We highlight the reproducibility of our workflow in Figure 4. Because the experiment depends on two key components, we rely on the reproducibility of CZE (Figure 4a,c) and SID (Figure 4b,d) individually. Specifically, the same protein complexes need to migrate out at almost the exact same time across runs. Two representative cases illustrate the near-exact migration times and similar structurally informative SID fragmentation patterns, highlighting the reproducibility of our Native MudPIT technique.

**Figure 4:**
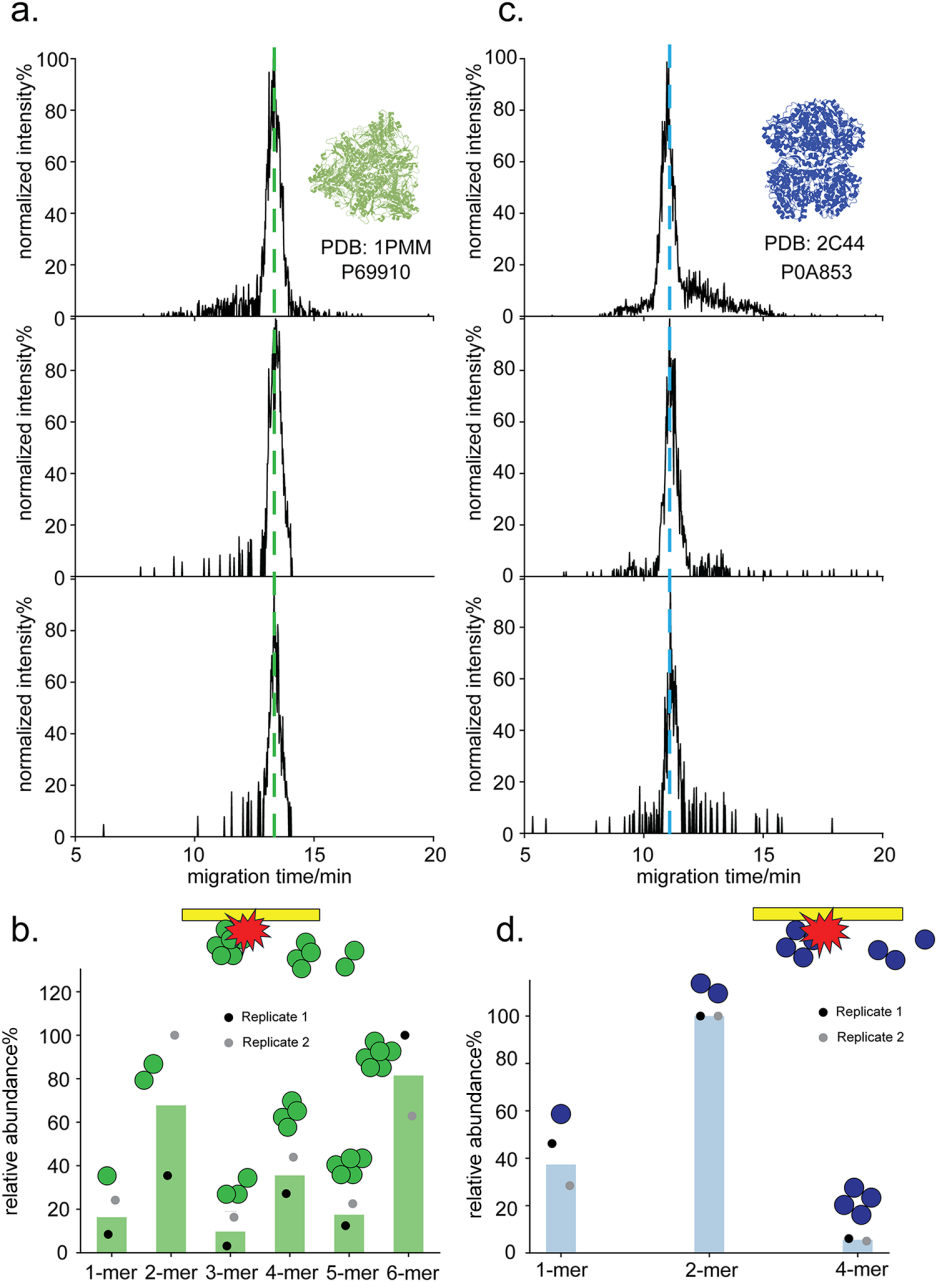
Highlighted examples illustrating the reproducibility of this method. (a). Representative extracted electropherogram for glutamate decarboxylase β in different CZE runs; (b). Deconvolved mass spectrum showing the abundance for each fragment of glutamate decarboxylase β with duplicates. Although the precursor to product distribution varies slightly, tetramer and dimer are more abundant than other fragments in both replicates, leading to the same structural conclusion (trimer of dimers). (c). Representative extracted electropherogram for tryptophanase in different CZE runs; (d). Deconvolved mass spectrum showing the abundance for each fragment of tryptophanase with duplicates.

We also noted that one recent study from the Sun group demonstrated the potential of label-free quantitative native proteomics using native CZE-MS to reveal the protein complex-level changes in a biological system under different conditions.^43^ Research leading to this manuscript and related follow-on experiments with other complex samples were generated in multiple locations, three times in the Wysocki lab via visits by the Sun lab to The Ohio State University, once by a Sun lab visit to Georgia Institute of Technology, now routinely and independently operated by both laboratories. A comparison of base peak electropherograms collected at different laboratory locations is shown in Figure S6. One of the main goals of the present work was to demonstrate the possibility of SID of complex biological samples at electrophoretic separation time scales.

The reproducible migration behavior and implementation across laboratories establishes that native structural proteomics is a robust and transferable workflow for probing protein complexes in complex biological samples, while systematic biological replication represents an important next step for quantitative biological studies.

## Conclusions

In conclusion, we have demonstrated a novel technology, Native MudPIT, for identifying protein complexes and effectively determining their topological structure directly from a complex biological system. Compared with our previous work, the current platform demonstrates improvements in coverage by ∼25%, in the scale of detected masses up to MDa ranges, and in the use of SID to characterize the topology of protein complexes directly from cell lysates.^8^ Our workflow successfully increased the effective separation space for protein complexes and enabled proteome-scale measurements of native assemblies using only nanogram amounts of material per run. Importantly, this moves native structural proteomics beyond assembly level mass-based detection alone and demonstrates the use of SID to characterize protein complexes in the context of a lysate. Together, these results highlight Native MudPIT as a promising first-generation platform for proteome-scale nMS measurements with access to stoichiometric and topological information.

As an early stage implementation, this platform points to clear opportunities for continued improvement. Although SID opens the door to fragmenting MDa and sub-MDa assemblies, generating sufficient fragment ions from large protein complexes within the CZE separation time scale remains an important technical frontier for high-confidence identification and topology characterization. Additionally, transient complexes and low-abundance species, like other methods, are still difficult to detect in complex biological samples, though we can detect a range of different complexes with a variety of protein classes. In the future, we foresee the incorporation of additional techniques such as enrichment to allow for detection of complexes that were not detected in the current workflow.

Future directions for further optimization include (1) incorporating quadrupole isolation and/or ion mobility spectrometry prior to SID, which could facilitate precursor selection, simplify species annotation, and improve confidence in assigning SID-derived subcomplex fragments. (2) Improvement in native SEC and CZE separation strategies, including but not limited to electrolyte concentrations, automation, sample loading and stacking, which could largely reduce artifactual modifications and enhance sensitivity, peak capacity, and the detection of transient species. (3) In parallel, continued development of dedicated processing workflows to reduce the need for manual interpretation of the data. With the recent development of new software tools, such as Proteoform Studio, we foresee that data processing can be more automated and streamlined.^58^ Together, these advances will continue to improve Native MudPIT as a robust platform for native structural proteomics.

Although the results shown here are for an *E. coli* lysate, work with human cells is in progress and giving promising results. Moreover, we see advantages to including ion mobility in this experiment, and are working on parallel experiments on an ion mobility QTOF platform. The longer-term goal is to develop instrumentation and protocols that would accomplish the SID composition/stoichiometry/topology experiments in the same instrument and workflow as the top-down proteomics (e.g., complex to subcomplex to top-down on monomers). This will not only further streamline Native MudPIT for more complex samples but also assign PTMs and sequence variants in the protein complexes directly. We foresee that Native MudPIT will be applied to a broad range of other biological systems, such as mammalian cells (and 2- and 3-D cell cultures), tissues, and biological fluids, e.g., with and without drug treatment or disease vs healthy, to advance our understanding of the biological functions of protein complex proteoforms (complexoforms)^2^ in modulating the fundamental biological processes and disease progression.

## Supplementary Material





## Data Availability

MS raw data and result files have been deposited to the ProteomeXchange Consortium via the PRIDE repository^59^ and are publicly accessible from its Web site with the data set identifier PXD069439.

## Notes

All voltages used for SID; mass spectra of the largest measured protein complex; mass spectra of tryptophanase in two SEC fractions; comparing HCD and SID for one protein complex; protein classification by different workflows; zoomed-in raw MS/MS of the monomer of glutamate decarboxylase β generated by either HCD or SID; base peak electropherogram of whole *E. coli* cell lysate at different location (PDF)
Spreadsheets containing the detected and identified lists of protein complexes, as well as the bottom-up and top-down proteomics data of each SEC fraction (XLSX)
