# Supplementary material for: Native Multidimensional Protein Complex Identification and Topology Characterization (Native MudPIT)
Source: Anal Chem. 2026 Jul 20;98(30):22165–75. doi: 10.1021/acs.analchem.6c01693 (PMC13449220; doi:10.1021/acs.analchem.6c01693)
Supplement: Supplementary file 1 [file ac6c01693_si_001.pdf]

Supporting Information I

Native multi-dimensional protein complex identification and topology characterization (Native MudPIT)

Zihao Qi[1,2] †, Fei Fang[3] †, William J Moeller[1,2] †, Guijie Zhu[3] †, Qianjie Wang[3], Qianyi Wang[3], Guangyao Gao[3], Liangliang Sun[3] *, Vicki H Wysocki[1,2] *

[1]School of Chemistry & Biochemistry, Georgia Institute of Technology, Atlanta, Georgia, 30332, United States.

[2]Native MS Guided Structural Biology Center, Georgia Institute of Technology, Atlanta, Georgia, 30332, United States.

[3]Department of Chemistry, Michigan State University, East Lansing, Michigan, 48824, United States.

† These authors contributed equally to this work.

Table of Contents

Supplementary Table S1
Table S1. .... S1
Supplementary Figure S2
Figure S1. .... S2
Figure S2. .... S3
Figure S3. .... S4
Figure S4. .... S5
Figure S5. .... S6
Figure S6. .... S7
References S8

Supplementary Table

Table S1. Voltage settings for SID experiments on the modified Q Exactive UHMR Orbitrap. The definition of SID voltage: the difference between the injection flatapole DC and the surface.

Lens	Transmission	SID 65 V	SID 125 V
Injection Flatapole (V)	5	5	5
Entrance 1-3 (V)	-6	-5	-5
Entrance 2 (V)	2	2	2
Front top (V)	0	-22.5	-19.5
Front bottom (V)	0	-8.5	-5.5
Surface (V)	0	-60	-120
Middle bottom (V)	0	-130	-190
Back top (V)	0	-174	-234
Back bottom (V)	0	-110	-170
Exit 1-3 (V)	3	-70	-130
Exit 2 (V)	0	-50	-110
C-trap entrance inject (V)	1.8	-49	-109
C-trap exit inject (V)	16	-40	-100
C-trap offset (V)	-5	-55	-115

Supplementary Figure

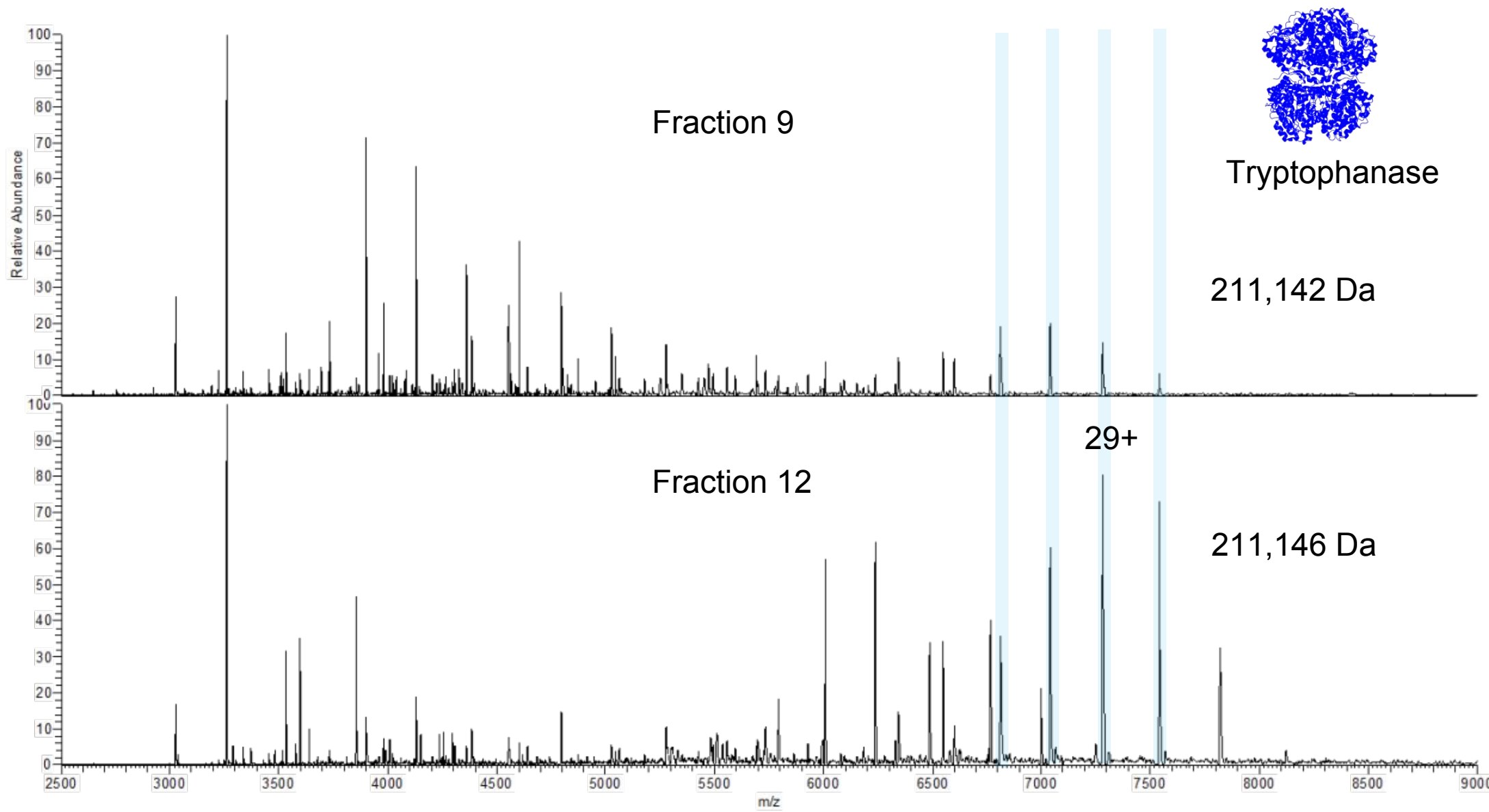

Figure S1. MS1 of a protein complex present in two fractions (fraction 9 and fraction 12; later identified as tryptophanase, see Figure.3d).

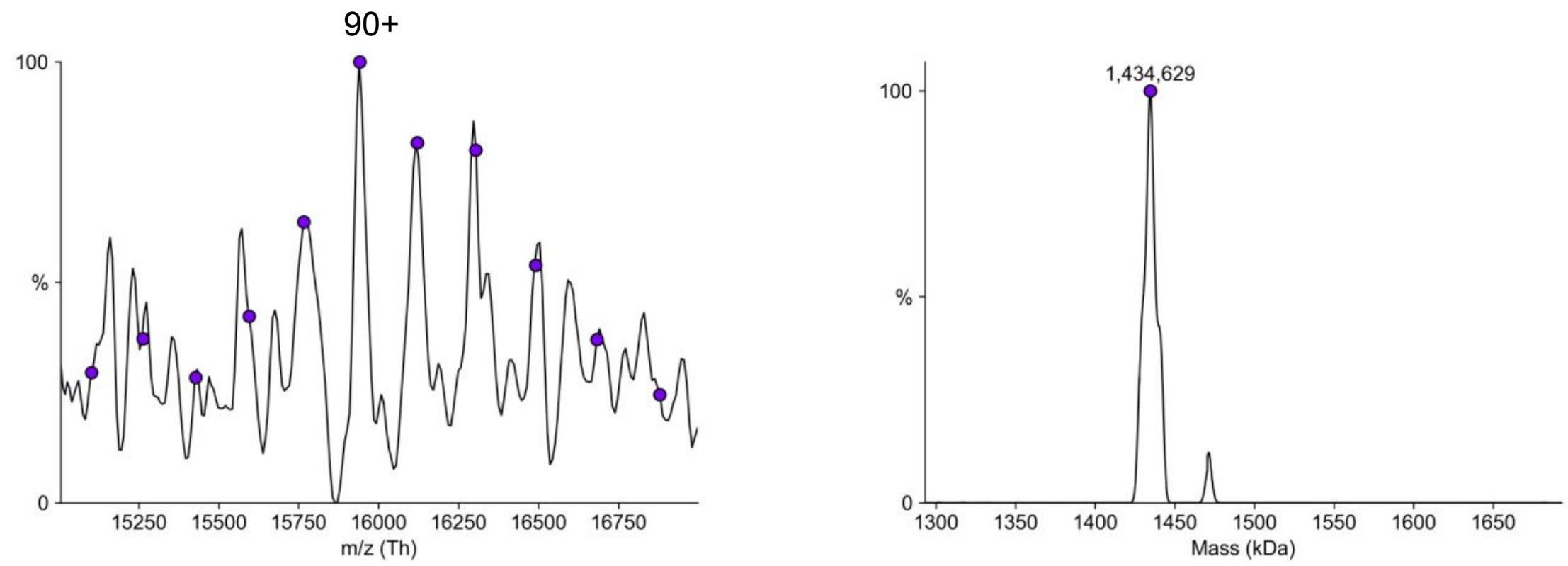

Figure S2. Mass spectrum and deconvolved mass spectrum of the 1.4 MDa species from fraction 5.

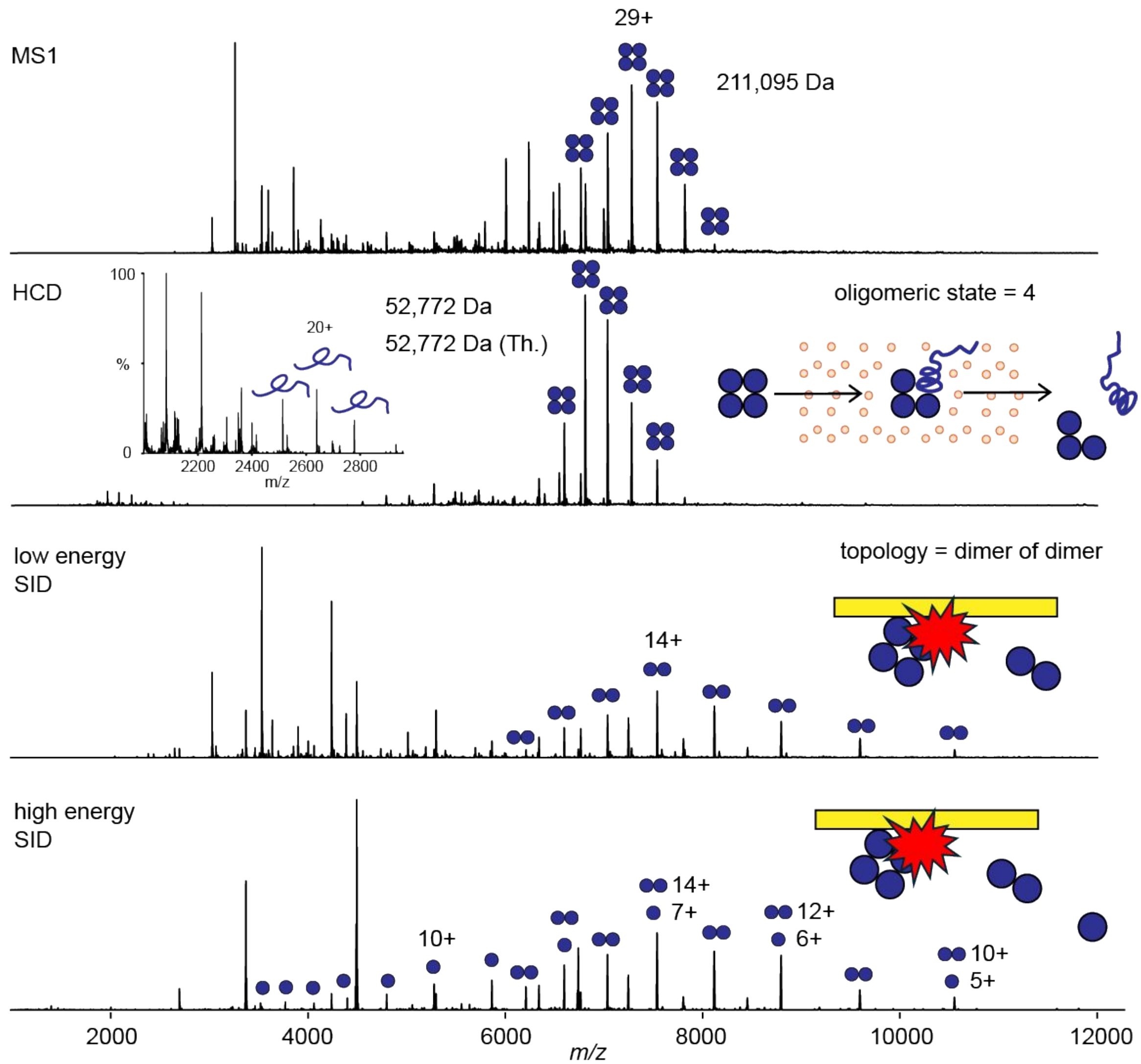

Figure S3. Comparing the HCD and SID spectra of one protein complex.

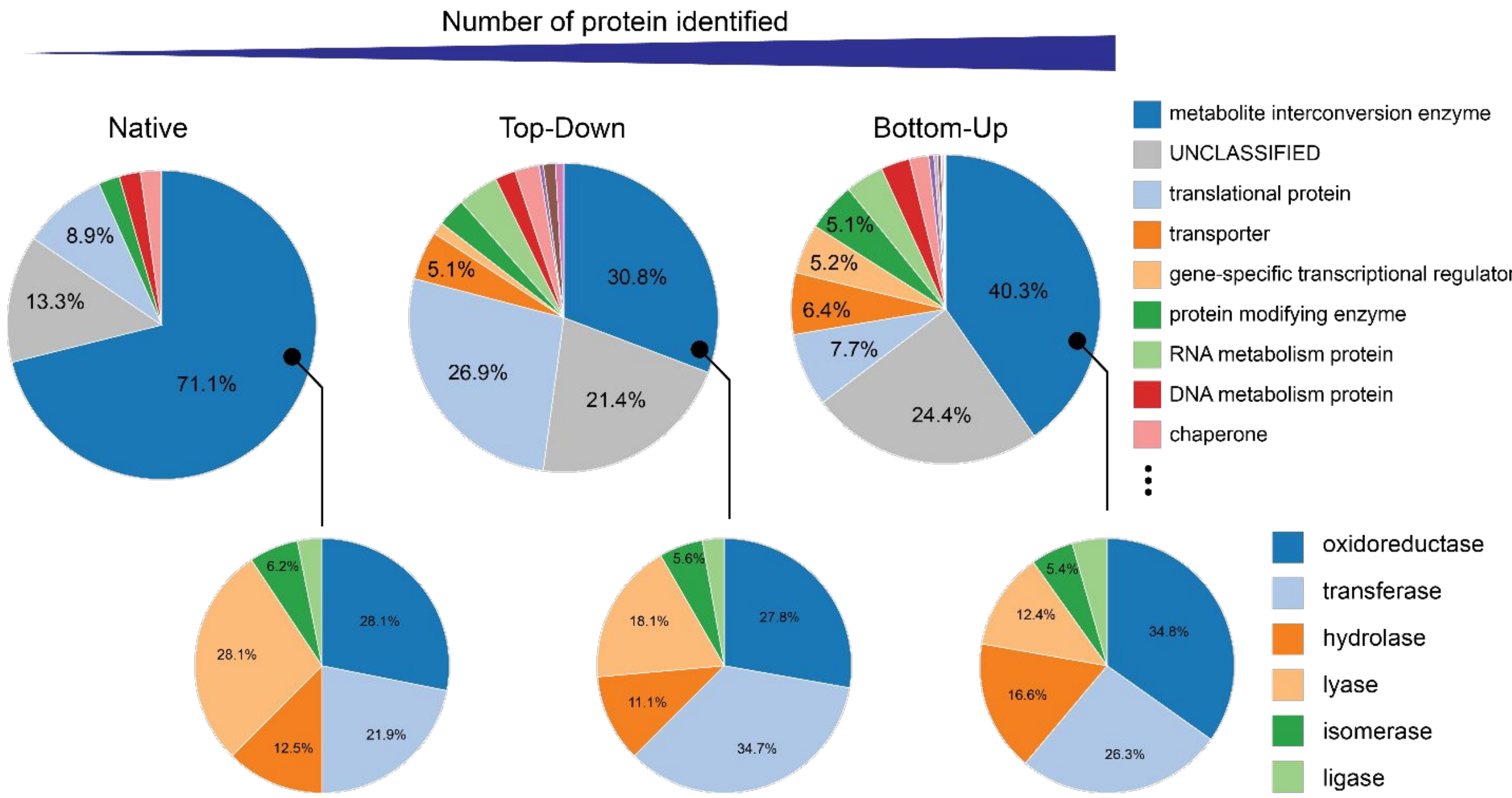

Figure S4. PANTHER Protein Class annotation of proteins identified by the three analytical workflows. Proteins identified by Native MudPIT, denaturing top-down proteomics, and bottom-up proteomics were classified using PANTHER Protein Class annotations. Pie charts summarize the broad protein classes represented by each workflow, with enzyme subclasses shown separately for metabolite interconversion enzymes (the primary class). The analysis was intended as a descriptive, proteomics-style overview, not as a statistical enrichment or pathway-level interpretation.[1]

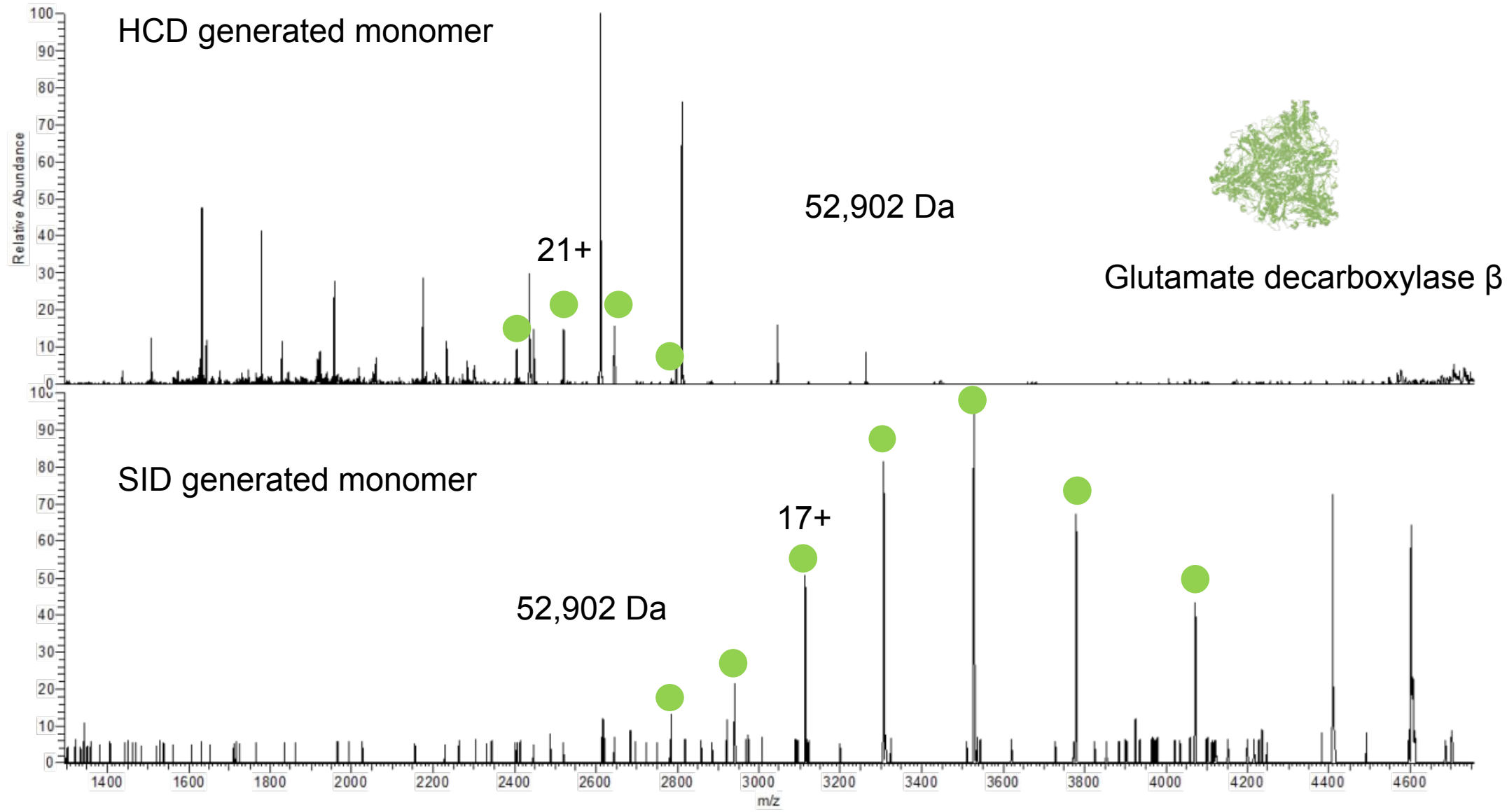

Figure S5. Zoomed-in raw MS/MS of the monomer for Glutamate decarboxylase β generated by either HCD (top) or SID (bottom)

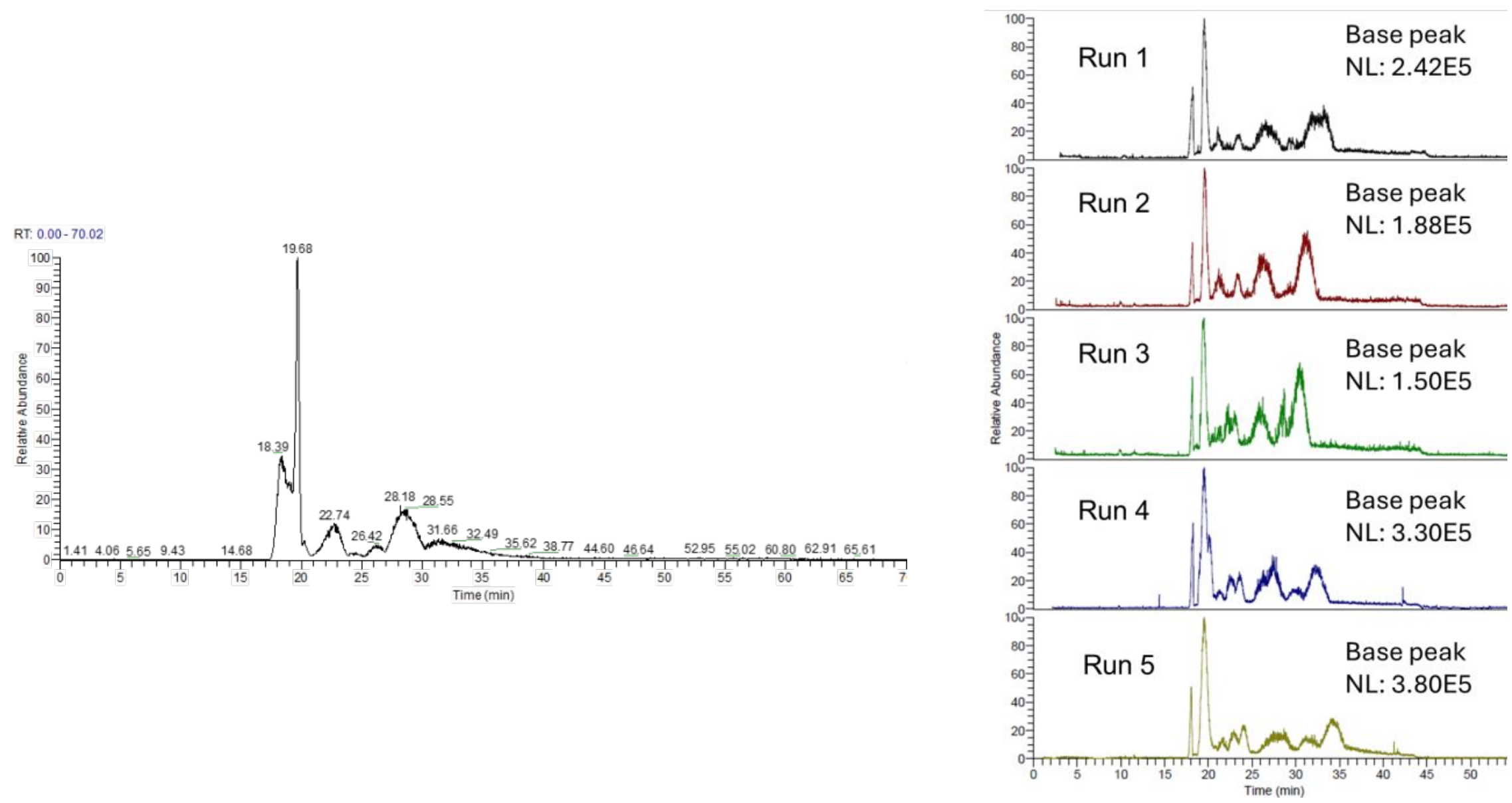

Figure S6. Base peak electropherograms comparison of the whole *E.coli* cell lysate using similar capillary (length, coating, etc.) and CE conditions at different locations. The goal of this figure is to demonstrate the robustness and reproducibility of this technique. (a) Base peak electropherogram adapted and reprocessed from Wang, Q. et al. Native Proteomics by Capillary Zone Electrophoresis-Mass Spectrometry. *Angew Chem Int Ed Engl* **2024**, e202408370, data was collected at The Ohio State University; (b) Base peak electropherogram adapted from Zhu, G. et al. Decades of Dreams Coming True: Capillary Zone Electrophoresis-Mass Spectrometry for Reproducible Multi-Level Proteomics. bioRxiv January 31, 2026, p 2026.01.28.702308, data was collected at Michigan State University.

References

(1) Mi, H.; Muruganujan, A.; Huang, X.; Ebert, D.; Mills, C.; Guo, X.; Thomas, P. D. Protocol Update for Large-Scale Genome and Gene Function Analysis with the PANTHER Classification System (v.14.0). *Nat Protoc* **2019**, *14* (3), 703–721. https://doi.org/10.1038/s41596-019-0128-8.
